# Phylogenetic Characterization of *Orthohantavirus dobravaense* (Dobrava Virus)

**DOI:** 10.3201/eid3004.230912

**Published:** 2024-04

**Authors:** Mert Erdin, Ceylan Polat, Teemu Smura, Sercan Irmak, Ortac Cetintas, Muhsin Cogal, Faruk Colak, Ahmet Karatas, Mustafa Sozen, Ferhat Matur, Olli Vapalahti, Tarja Sironen, Ibrahim Mehmet Ali Oktem

**Affiliations:** University of Helsinki, Helsinki, Finland (M. Erdin, T. Smura, O. Vapalahti, T. Sironen);; Hacettepe University, Ankara, Turkey (C. Polat); Balıkesir University, Balıkesir, Turkey (S. Irmak);; Bulent Ecevit University, Zonguldak, Turkey (O. Cetintas, M. Cogal, F. Colak, M. Sozen);; Ömer Halisdemir University, Niğde, Turkey (A. Karatas);; Dokuz Eylul University, Izmir, Turkey (F. Matur, I.M.A. Oktem)

**Keywords:** Orthohantavirus dobravaense, Dobrava virus, orthohantaviruses, viruses, zoonoses, airborne pathogens, phylogeny, epidemiology, European Union, European Economic Area

## Abstract

We report complete coding sequences of *Orthohantavirus dobravaense* (Dobrava virus) Igneada strains and phylogenetic characterization of all available complete coding sequences. Our analyses suggested separation of host-dependent lineages, followed by geographic clustering. Surveillance of orthohantaviruses using complete genomes would be useful for assessing public health threats from Dobrava virus.

Orthohantaviruses are globally distributed. Until now, they have been detected in rodents, insectivores, and bats. Rodentborne orthohantaviruses, which are associated with human diseases, are divided into 3 major groups, murid-borne, non–*Arvicolinae cricetidae*-borne, and *Arvicolinae*-borne viruses, according to their phylogeny and host species (*1*). Murid-borne orthohantavirus species, such as *Orthohantavirus dobravaense* (Dobrava virus; DOBV) and *O. hantanense* (Hantaan virus), which are associated with hemorrhagic fever with renal syndrome in humans, are distributed in the Old World (*1*,*2*). Non–*A. cricetidae*-borne orthohantaviruses, such as *O. bayoui* (Bayou virus) or *O. sinnombreense* (Sin Nombre virus), which cause hantavirus cardiopulmonary syndrome in human infections, are found in the Americas (*1*,*2*). *Arvicolinae*-borne orthohantaviruses, such as *O. puumalaense* (Puumala virus; PUUV) or *O. prospectense* (Prospect Hill virus), are either nonpathogenic or mildly pathogenic for humans (*1*,*2*) and are found in both the Old and New Worlds; *Arvicolinae*-borne strains are thought to serve as an evolutionary bridge between the other 2 groups. 

Orthohantaviruses can be transmitted to humans through inhalation of virus-containing aerosols of rodent excreta or direct contact with reservoir hosts (*2*). In European Union/European Economic Area countries, the numbers of collective orthohantavirus case reports fluctuated between 1,647 and 4,249 cases during 2016–2020 (*3*). For instance, in 2020, PUUV virus caused 1,204 cases, Hantaan virus 14 cases, and DOBV 7 cases from the reports that confirmed laboratory information available for the causative viruses. The highest number of cases of hemorrhagic fever with renal syndrome have been detected in southeastern Europe, with 2,375 cases reported in the Balkan region during 1952–2012, most caused by PUUV or DOBV (*3*,*4*). DOBV-positive rodents have recently been found in northeastern Italy, suggesting potential geographic expansion of this clade (*5*). 

Surveillance studies in rodent populations are essential for understanding the dynamics of fluctuations. Earlier studies have shown that geographic barriers might play a role in genetic diversity and clade separation among DOBV (*6*,*7*). Also, obtaining whole-genome sequences is a crucial step in understanding potential viral genetic determinants of phenotypic changes that might affect disease severity among these viruses. We report complete coding sequences of *O. dobravaense* Igneada strain and phylogenetic characterization of all available complete coding sequences of DOBV. 

## The Study

DOBV has caused human cases and outbreaks in the northern coastal region of Turkey (*8*–*12*). In a previous study, DOBV seropositivity and RNA positivity were discovered in rodents captured in Kirklareli Province in Eastern Thrace in Turkey, and phylogenetic analysis based on partial DOBV genomes suggested that DOBV strains from Igneada, Turkey, are closely related to strains from Balkan countries (*13*). To understand the phyloepidemiologic distribution of DOBV, we sequenced complete coding regions of DOBV Igneada strains (GenBank accession nos. MW055917–9) from 1 archived sample that had been partially sequenced in a previous study (*13*); we compared results from the phylogenetic analyses with all available complete DOBV coding sequences in GenBank. Because of the limited number (n = 16) of complete DOBV coding sequences for all 3 segments currently available in GenBank, in addition to 55 complete small (S), 25 medium (M), and 16 large (L) sequences, we also analyzed a larger dataset of partial S-segment sequences (Appendix). 

**Figure 1:**
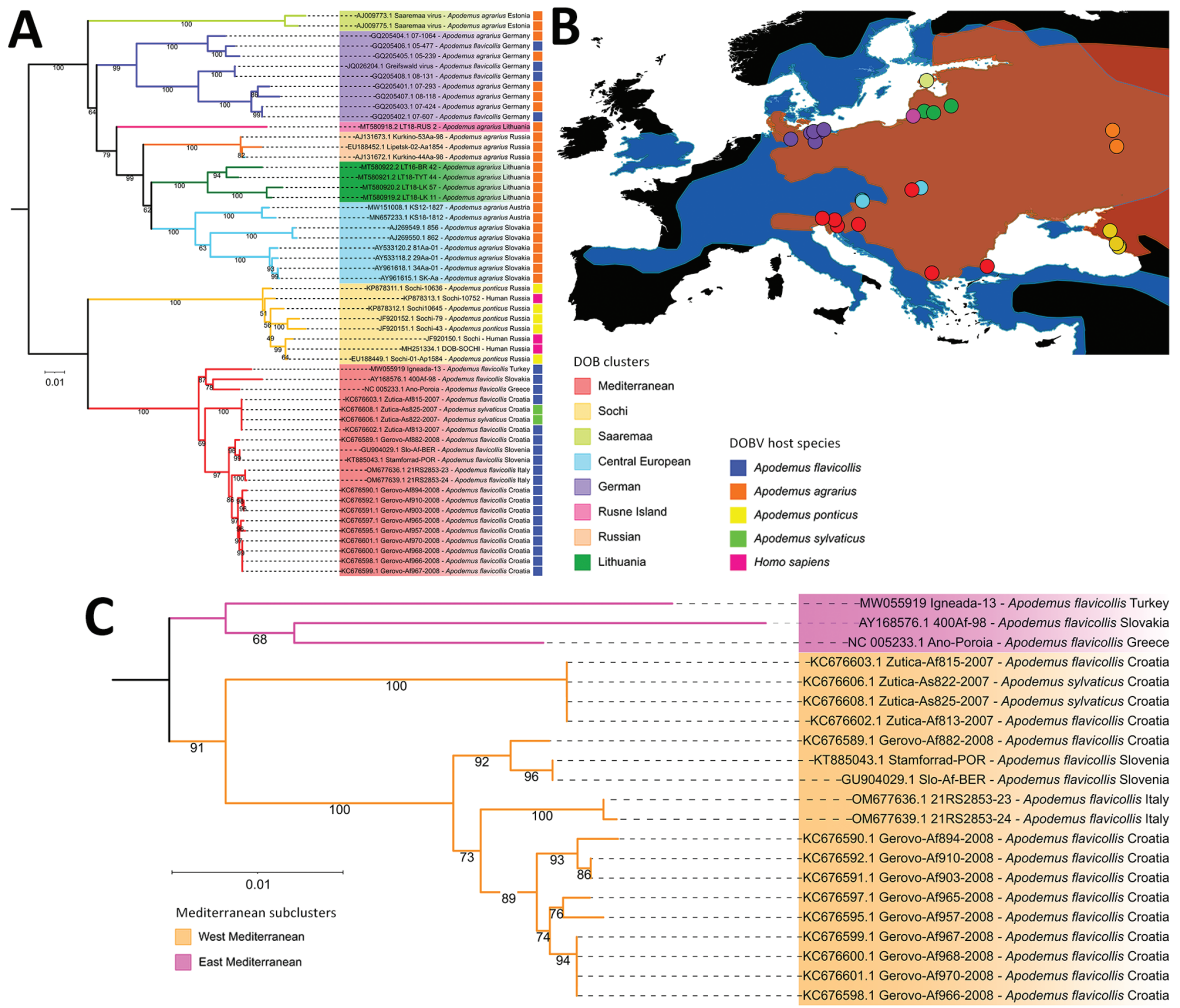
Phylogenetic characterization of DOBV combined with reservoir host and geographical distribution data. A) Maximum-likelihood tree based on all available complete DOBV sequences constructed using a transition plus empirical base frequencies plus gamma 4 substitution model. Colors indicate major clusters and hosts from which sequences were obtained. B) Distribution map of 2 major DOBV reservoir hosts, *Apodemus flavicollis* (blue) and *A. agrarius* (orange) mice, and their overlapping distribution zones. Solid circles indicate locations of complete sequences used in maximum-likelihood tree . C) Pruned version of the tree in panel A showing the division of the Mediterranean cluster into West and East Mediterranean subclusters. DOBV, Dobrava virus (*Orthohantavirus dobravaense*).

Phylogenetic analyses (Figure 1, panel A; Appendix Figure 1) and pairwise nucleotide identities (Appendix Figure 2) suggested 8 major clusters, designated by their main distribution ranges: Mediterranean, Sochi, Saaremaa, Central Europe, Germany, Rusne Island, Lithuania, and Russia. Consistent with a previous study (*13*), the DOBV Igneada strains sequenced in this study grouped together with strains from the cluster from the Mediterranean region. Of note, most human DOBV cases were from this region (*14*). The Mediterranean clade is further regionally separated into West and East Mediterranean subclades (Figure 1, panel C). The West Mediterranean subclade consists of strains from Italy, Slovenia, Croatia, Hungary, and Kosovo; the East Mediterranean subclade comprises strains from Turkey, Greece, and eastern Slovakia (Appendix Figure 3). 

**Figure 2:**
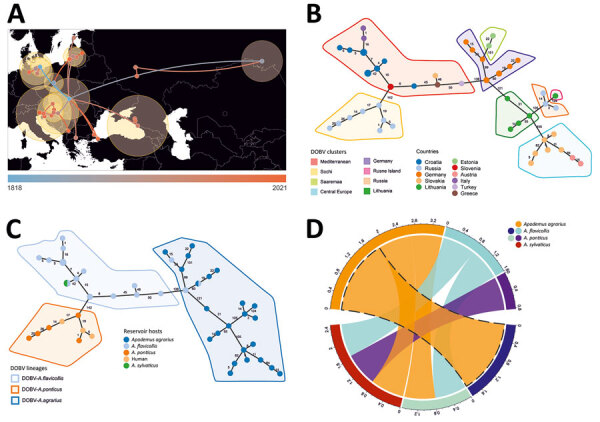
Host switching, phylogeographic reconstruction, and phylogenetic characterization of DOBV according to Bayesian analysis and minimum spanning tree constructions. A) Phylogeographic reconstruction of DOBV in discrete space. Each node is colored according to the estimated year of discovery, from the earliest (blue) to the latest (orange). Yellow shaded circles show the relative intensity of local viruses spread in the covered area. B) Minimum spanning tree showing the geographical cluster separation. C) Minimum spanning tree suggesting 3 major lineages according to reservoir host species. D) Chord diagram representing host switching rates of DOBV between 4 rodent species: *Apodemus flavicollis* (yellow-necked mouse), *A. agrarius* (striped field mouse), *A. ponticus* (Black Sea field mice), and *A. sylvaticus* (wood mice). DOBV, Dobrava virus (*Orthohantavirus dobravaense*).

Bayesian phylogeographic reconstruction based on all available complete and partial (>750 bases) S-segment sequences suggested that the estimated root location of DOBV is in Slovakia and Hungary in eastern Europe; from there, the virus has spread to other regions through multiple introductions, followed by local spreading (Figure 2, panel A). Minimum spanning tree phylogeny showing clear geographic clustering also supports that supposition (Figure 2, panel B). It should be noted, however, that sequence data are lacking for wide areas within the potential geographic distribution range of the main hosts of DOBV, and further studies are needed in those areas to confirm initial findings of clustering. 

We derived DOBV sequences from 4 host species: *Apodemus flavicollis* (yellow-necked mice), *A. agrarius* (striped field mice), *A. sylvaticus* (wood mice), and *A. ponticus* (Black Sea field mice)*.* Consistent with earlier studies (*15*), topology in the DOBV phylogenetic tree correlates with the geographic ranges of host species (Figure 1, panel A). Bayesian analysis suggested host-dependent lineage separation, followed by geographic clustering (Appendix Figure 3). The minimum spanning phylogenic tree correlated with the Bayesian analysis in showing clear host-dependent separation (Figure 2, panel C). In addition, our analysis suggested host-switching events between *A. flavicollis* and *A. agrarius* mice (Figure 2, panel D). The distribution ranges of *A. flavicollis* and *A. agrarius* mice overlap in eastern Europe and some parts of central Europe. In northern Germany, there is a close phylogenetic relation of DOBV strains with those 2 reservoir hosts (Figure 1, panel B). Although probability estimates in our analysis did not support host-switching between the other host species, that lack of information might have resulted from lack of sufficient sequence data, especially on potential host-switching or spillover events between *A. flavicollis* and *A. sylvaticus* mice (Figure 1, panel A; Appendix Figure 3). 

## Conclusions

Tracking viral genetic changes using complete genome sequences to characterize viruses circulating in rodent populations is a crucial first step for understanding the spatiotemporal epidemiologic patterns of orthohantavirus-induced diseases and potential viral genetic determinants of virulence. Phylogenetic characterization of DOBV strains according to geographic regions within Europe and bordering countries suggests that more thorough genomic surveillance of orthohantaviruses, preferably using complete genomes, would be useful for assessing the DOBV-induced threat to public health. 

Appendix Additional information about phylogenetic characterization of *O Orthohantavirus dobravaense* virus (Dobrava virus).
