# Supplementary material for: Phylogenetic Characterization of Orthohantavirus dobravaense (Dobrava Virus)
Source: Emerg Infect Dis. 2024 Apr;30(4):779–82. doi: 10.3201/eid3004.230912 (PMC10977844; doi:10.3201/eid3004.230912)
Supplement: Appendix — Additional information about phylogenetic characterization of O Orthohantavirus dobravaense virus (Dobrava virus). [file 23-0912-Techapp-s1.pdf]

Article DOI: https://doi.org/10.3201/eid3004.230912

EID cannot ensure accessibility for Appendix materials supplied by authors. Readers who have difficulty accessing Appendix content should contact the authors for assistance.

Phylogenetic Characterization of *Orthohantavirus dobravaense* (Dobrava Virus)

Appendix

Material and Methods

In earlier study, we obtained partial sequences of each segment of DOBV from Igneada region in Turkey (*1*). We used one of those sample in our complete genome sequencing. We used archived RNA extracted by using Invitrogen TRIzol (Thermo Fisher Scientific, https://www.thermofisher.com) following the manufacturer's guidelines. We used NEBNext rRNA depletion kit (human/mouse/rat) to remove host rRNA, and NEBNext Ultra II RNA library preparation kit (New England Biolabs, https://www.neb.com) to construct the sequencing library. We performed next-generation sequencing (NGS) using Illumina MiSeq system. We quality-filtered and de-novo assembled the raw data and annotated the contigs with LazyPipe (*2*). We filled the gaps in the sequences by designing primers to the genomic regions flanking the gaps (Appendix Table), performing polymerase chain reaction (PCR). These amplicons were sequenced by the Sanger method. We aligned our sequences for each segment separately with all available DOBV complete coding sequences of each encoded protein retrieved from the GenBank using ClustalW algorithm implemented in MegaX software and constructed maximum likelihood (ML) trees using IQ-TREE2 (http://www.iqtree.org) and ModelFinder for the best-fitted model for tree construction. We used PHYLOVIZ (https://www.phyloviz.net) for minimum spanning tree construction to support cluster hypothesis. We calculated pairwise identities from nucleotide sequences by using Sequence Demarcation Tool version 1.2 (University of Cape Town, http://web.cbio.uct.ac.za/~brejnev). For the phylogeographic reconstruction, host switching estimates, and Bayesian time tree construction of S segment, we used BEAST v1.10.4. In the BEAST analysis, the dataset included both complete coding

sequences and partial sequences which were equal to or longer than 750 nt and ended up total of 127 taxa for phylogeography and 107 taxa for host switching (DOBV sequences from human samples were excluded) analyses. Analysis parameters were as follows: tip dates enabled, host and geographic locations as discreet traits, uncorrelated relaxed clock for molecular clock, codon partition for each nucleotide separately to provide unique evolutionary rate for each position under Tamura-Nei 93 model with gamma categories as 5 and with invariant sites, population prior assumption to be constant, Markov chain Monte Carlo (MCMC) length to be 376.69×10^6 and echo sampling in every 10000 for phylogeographic reconstruction, and MCMC length to be 3.5×10^8 and echo sampling in every 1000 for host switching estimation. We used Tracer v1.7.2 to evaluate MCMC convergence for effective sample size to be >200 for each parameter. Bayesian trees were annotated to maximum clade credibility tree in TreeAnnotator v1.10.4. We used Spread3 v0.9.7.1 for the visualization of discreet phylogeographic reconstruction. Likelihood mapping assessment were done by IQ-TREE2 as 10000 quartets, and molecular saturation were extracted by DAMBE software with general time reversible distances. Data visualization was done in R v4.3.1/R studio. In earlier studies, it was hypothesized that recombination between DOBV strains may occur in nature (*3*). Thus, we tested our dataset for each segment with RDP version 5.30 (University of Cape Town, http://web.cbio.uct.ac.za/~darren/rdp.html) with all recombination testing methods implemented in this software package.

Appendix References

1. Polat C, Sironen T, Plyusnina A, Karatas A, Sozen M, Matur F, et al. *Dobrava hantavirus* variants found in *Apodemus flavicollis* mice in Kırklareli Province, Turkey. J Med Virol. 2018;90:810–8. PubMed https://doi.org/10.1002/jmv.25036

2. Plyusnin I, Kant R, Jaaskelainen AJ, Sironen T, Holm L, Vapalahti O, et al. Novel NGS pipeline for virus discovery from a wide spectrum of hosts and sample types. Virus Evol. 2020;6:veaa091.

3. Klempa B, Schmidt HA, Ulrich R, Kaluz S, Labuda M, Meisel H, et al. Genetic interaction between distinct *Dobrava hantavirus* subtypes in *Apodemus agrarius* and *A. flavicollis* in nature. J Virol. 2003;77:804–9. PubMed https://doi.org/10.1128/JVI.77.1.804-809.2003

Appendix Table. List of designed primers to fill the gaps on the DOBV Igneada strain sequences by PCR and Sanger sequencing.

Primer	Sequence	Segment	Length
Forward_S_(G1)	ACAACCACGAAGGCCAACTG	S	20
Reverse_S_(G1)	TGTCCTGTAGTCTCATCAATGTC	S	23
Forward_S_(G2)	GATATGAGGAATACCATCATGGC	S	23
Reverse_S_(G2)	CCTAGTGCAAATACATCCACCAA	S	23
Forward_M_(G1)	GAGACAACATCAAGTGAGGTCAA	M	23
Reverse_M_(G1)	GAAACAATCCTGGGCTATAAACG	M	23
Forward_M_(G2)	GGTGTTACCGGACATTAAATCTC	M	23
Reverse_M_(G2)	CAGGATTACAGCCCCAACTG	M	20
Forward_L_(G1)	GAGGGATTGGTTATCAAAAAGCC	L	23
Reverse_L_(G1)	GTGGGTTCACTTATATTGAGCTC	L	23
Forward_L_(G2)	CGAAGTCTCAGGTTGTAGCTAA	L	22
Reverse_L_(G2)	GTTCAATAAAGCTCTCCCCAGA	L	22
Forward_L_(G3)	GAAGGCTGTGCTGTATCAATAC	L	22
Reverse_L_(G3)	TGCATGTAACCTAAAAGTGCC	L	21
Forward_L_(G4)	GAGGTAACTCAAGAAGATCTTG	L	22
Reverse_L_(G4)	GAAGGTCACCTTCATAGAGC	L	20
Forward_L_(G5)	CCCCTGCTGCATACTCATTAA	L	21
Reverse_L_(G5)	CCTTTTTGAGATACCAGAAGCT	L	22

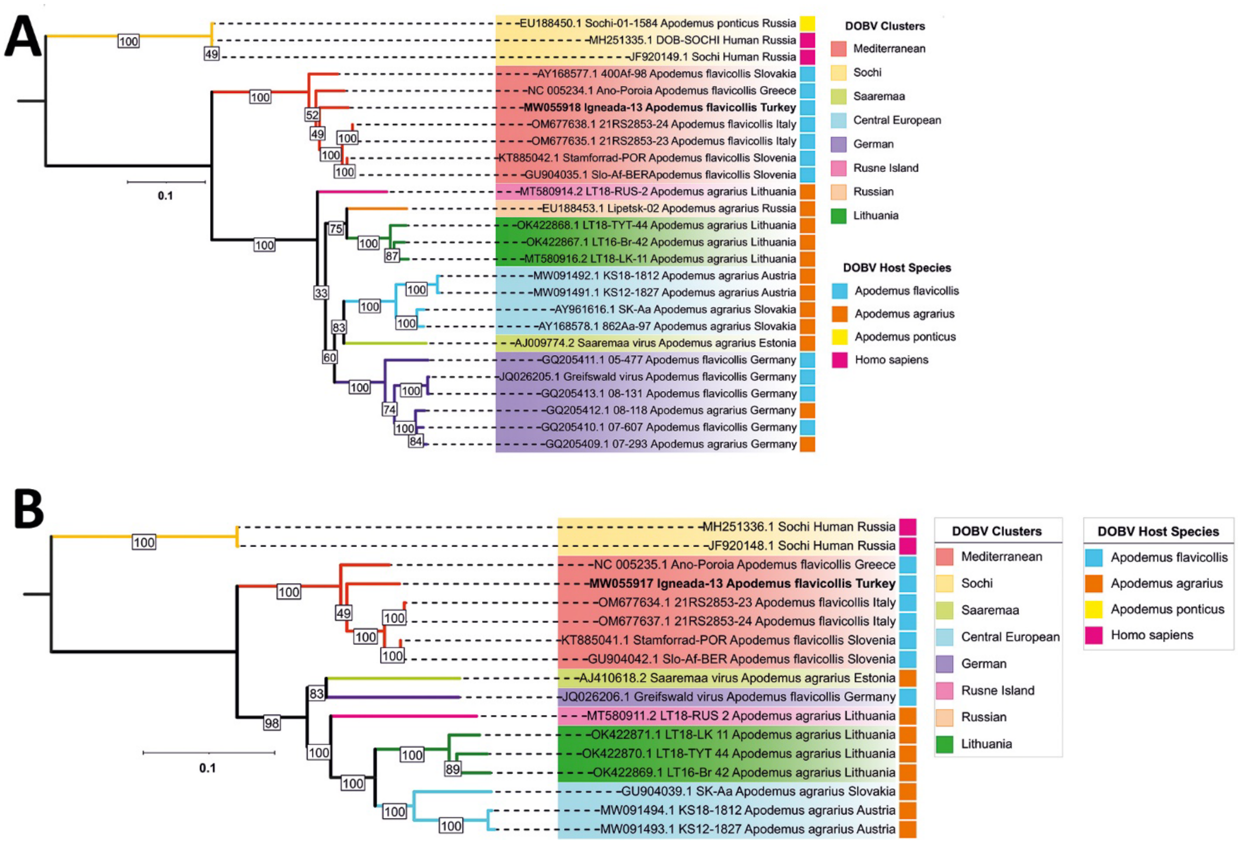

Appendix Figure 1. Phylogenetic characterization of DOBV combined with reservoir host and geographical distribution data. Maximum likelihood trees of A) M-segment based on all available complete DOBV sequences constructed using TIM2+F+I+R2 showed geographic clustering, and B) of L-segment based on all available complete DOBV sequences constructed using GTR+F+I+R2 showed geographical clustering in correlation with S and M segments.

A

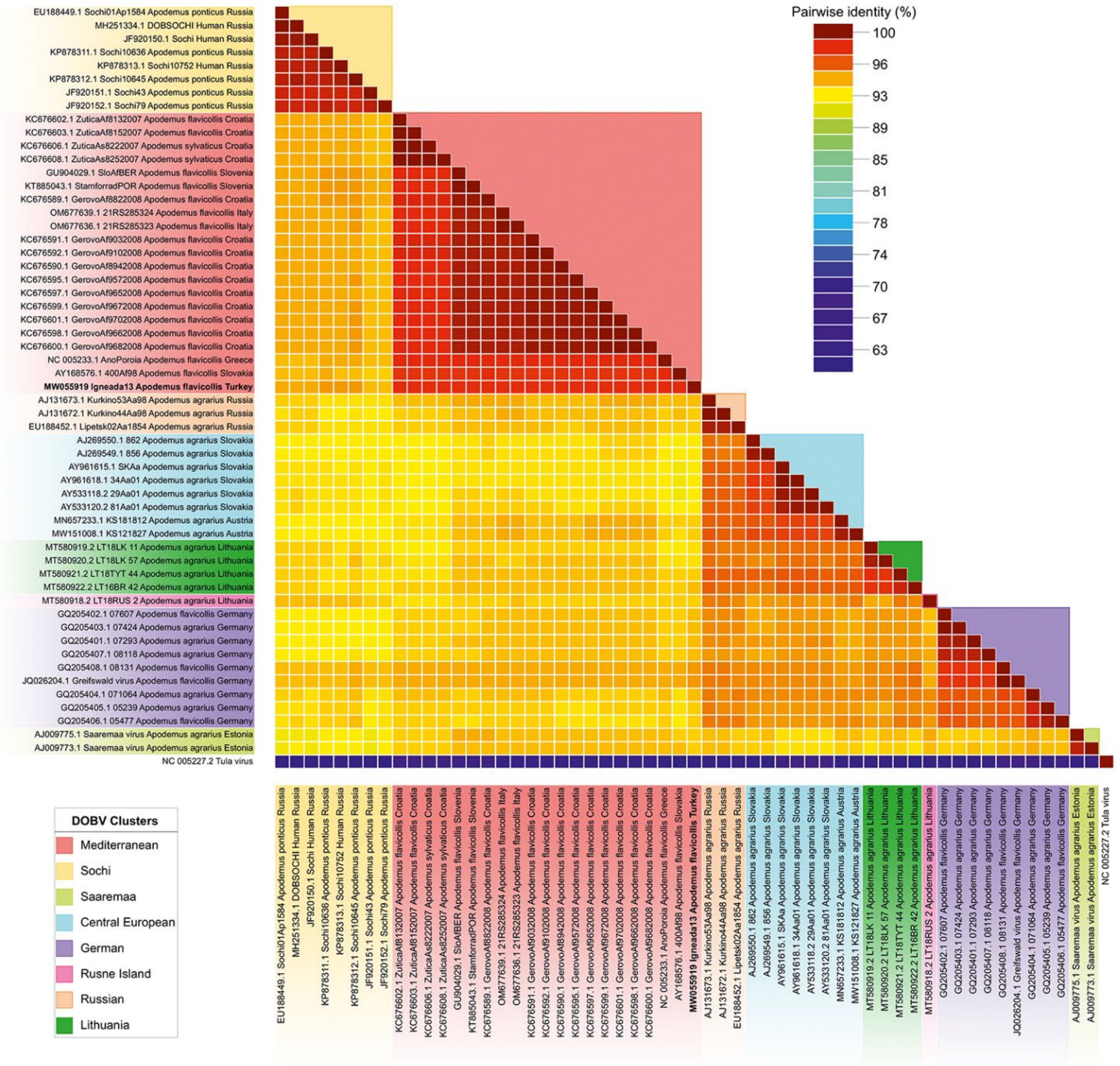
Pairwise identity (%)
100
96
93
89
85
81
78
74
70
67
63
EU188449.1 Sochi01Ap1584 Apodemus ponticus Russia
MH251334.1 DOBSOCHI Human Russia
JF920150.1 Sochi Human Russia
KP878311.1 Sochi10636 Apodemus ponticus Russia
KP878313.1 Sochi10752 Human Russia
KP878312.1 Sochi10645 Apodemus ponticus Russia
JF920151.1 Sochi43 Apodemus ponticus Russia
JF920152.1 Sochi79 Apodemus ponticus Russia
KC676602.1 ZuticaAf8132007 Apodemus flavicollis Croatia
KC676603.1 ZuticaAf8152007 Apodemus flavicollis Croatia
KC676606.1 ZuticaAs8222007 Apodemus sylvaticus Croatia
KC676608.1 ZuticaAs8252007 Apodemus sylvaticus Croatia
GU904029.1 SloAfBER Apodemus flavicollis Slovenia
KT885043.1 StamforradPOR Apodemus flavicollis Slovenia
KC676589.1 GerovoAf8822008 Apodemus flavicollis Croatia
OM677639.1 21RS285324 Apodemus flavicollis Italy
OM677636.1 21RS285323 Apodemus flavicollis Italy
KC676591.1 GerovoAf9032008 Apodemus flavicollis Croatia
KC676592.1 GerovoAf9102008 Apodemus flavicollis Croatia
KC676590.1 GerovoAf8942008 Apodemus flavicollis Croatia
KC676595.1 GerovoAf9572008 Apodemus flavicollis Croatia
KC676597.1 GerovoAf9652008 Apodemus flavicollis Croatia
KC676599.1 GerovoAf9672008 Apodemus flavicollis Croatia
KC676601.1 GerovoAf9702008 Apodemus flavicollis Croatia
KC676598.1 GerovoAf9662008 Apodemus flavicollis Croatia
KC676600.1 GerovoAf9682008 Apodemus flavicollis Croatia
NC 005233.1 AnoPoroia Apodemus flavicollis Greece
AY168576.1 400Af98 Apodemus flavicollis Slovakia
MW055919 Igneada13 Apodemus flavicollis Turkey
AJ131673.1 Kurkino53Aa98 Apodemus agrarius Russia
AJ131672.1 Kurkino44Aa98 Apodemus agrarius Russia
EU188452.1 Lipetsk02Aa1854 Apodemus agrarius Russia
AJ269550.1 862 Apodemus agrarius Slovakia
AJ269549.1 856 Apodemus agrarius Slovakia
AY961615.1 SKAa Apodemus agrarius Slovakia
AY961618.1 34Aa01 Apodemus agrarius Slovakia
AY533118.2 29Aa01 Apodemus agrarius Slovakia
AY533120.2 81Aa01 Apodemus agrarius Slovakia
MN657233.1 KS181812 Apodemus agrarius Austria
MW151008.1 KS121827 Apodemus agrarius Austria
MT580919.2 LT18LK 11 Apodemus agrarius Lithuania
MT580920.2 LT18LK 57 Apodemus agrarius Lithuania
MT580921.2 LT18TYT 44 Apodemus agrarius Lithuania
MT580922.2 LT16BR 42 Apodemus agrarius Lithuania
MT580918.2 LT18RUS 2 Apodemus agrarius Lithuania
GQ205402.1 07607 Apodemus flavicollis Germany
GQ205403.1 07424 Apodemus agrarius Germany
GQ205401.1 07293 Apodemus agrarius Germany
GQ205407.1 08118 Apodemus agrarius Germany
GQ205408.1 08131 Apodemus flavicollis Germany
JQ026204.1 Greifswald virus Apodemus flavicollis Germany
GQ205404.1 071064 Apodemus agrarius Germany
GQ205405.1 05239 Apodemus agrarius Germany
GQ205406.1 05477 Apodemus flavicollis Germany
AJ009775.1 Saaremaa virus Apodemus agrarius Estonia
AJ009773.1 Saaremaa virus Apodemus agrarius Estonia
NC 005227.2 Tula virus
DOBV Clusters
Mediterranean
Sochi
Saaremaa
Central European
German
Rusne Island
Russian
Lithuania

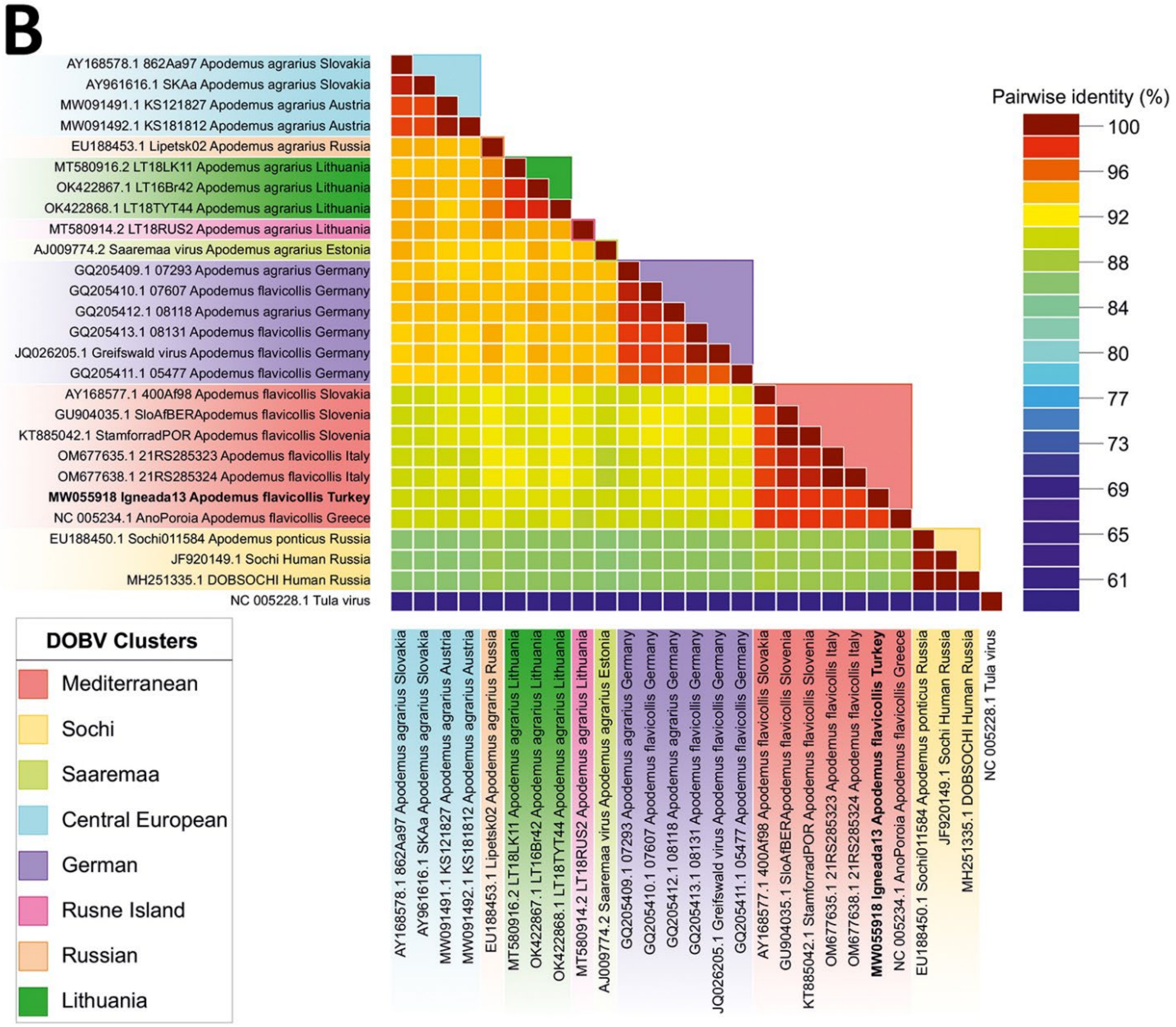
B
AY168578.1 862Aa97 Apodemus agrarius Slovakia
AY961616.1 SKAa Apodemus agrarius Slovakia
MW091491.1 KS121827 Apodemus agrarius Austria
MW091492.1 KS181812 Apodemus agrarius Austria
EU188453.1 Lipetsk02 Apodemus agrarius Russia
MT580916.2 LT18LK11 Apodemus agrarius Lithuania
OK422867.1 LT16Br42 Apodemus agrarius Lithuania
OK422868.1 LT18TYT44 Apodemus agrarius Lithuania
MT580914.2 LT18RUS2 Apodemus agrarius Lithuania
AJ009774.2 Saaremaa virus Apodemus agrarius Estonia
GQ205409.1 07293 Apodemus agrarius Germany
GQ205410.1 07607 Apodemus flavicollis Germany
GQ205412.1 08118 Apodemus agrarius Germany
GQ205413.1 08131 Apodemus flavicollis Germany
JQ026205.1 Greifswald virus Apodemus flavicollis Germany
GQ205411.1 05477 Apodemus flavicollis Germany
AY168577.1 400Af98 Apodemus flavicollis Slovakia
GU904035.1 SloAfBERApodemus flavicollis Slovenia
KT885042.1 StamforradPOR Apodemus flavicollis Slovenia
OM677635.1 21RS285323 Apodemus flavicollis Italy
OM677638.1 21RS285324 Apodemus flavicollis Italy
MW055918 Igneada13 Apodemus flavicollis Turkey
NC 005234.1 AnoPoroia Apodemus flavicollis Greece
EU188450.1 Sochi011584 Apodemus ponticus Russia
JF920149.1 Sochi Human Russia
MH251335.1 DOBSOCHI Human Russia
NC 005228.1 Tula virus
Pairwise identity (%)
100
96
92
88
84
80
77
73
69
65
61
DOBV Clusters
Mediterranean
Sochi
Saaremaa
Central European
German
Rusne Island
Russian
Lithuania

C

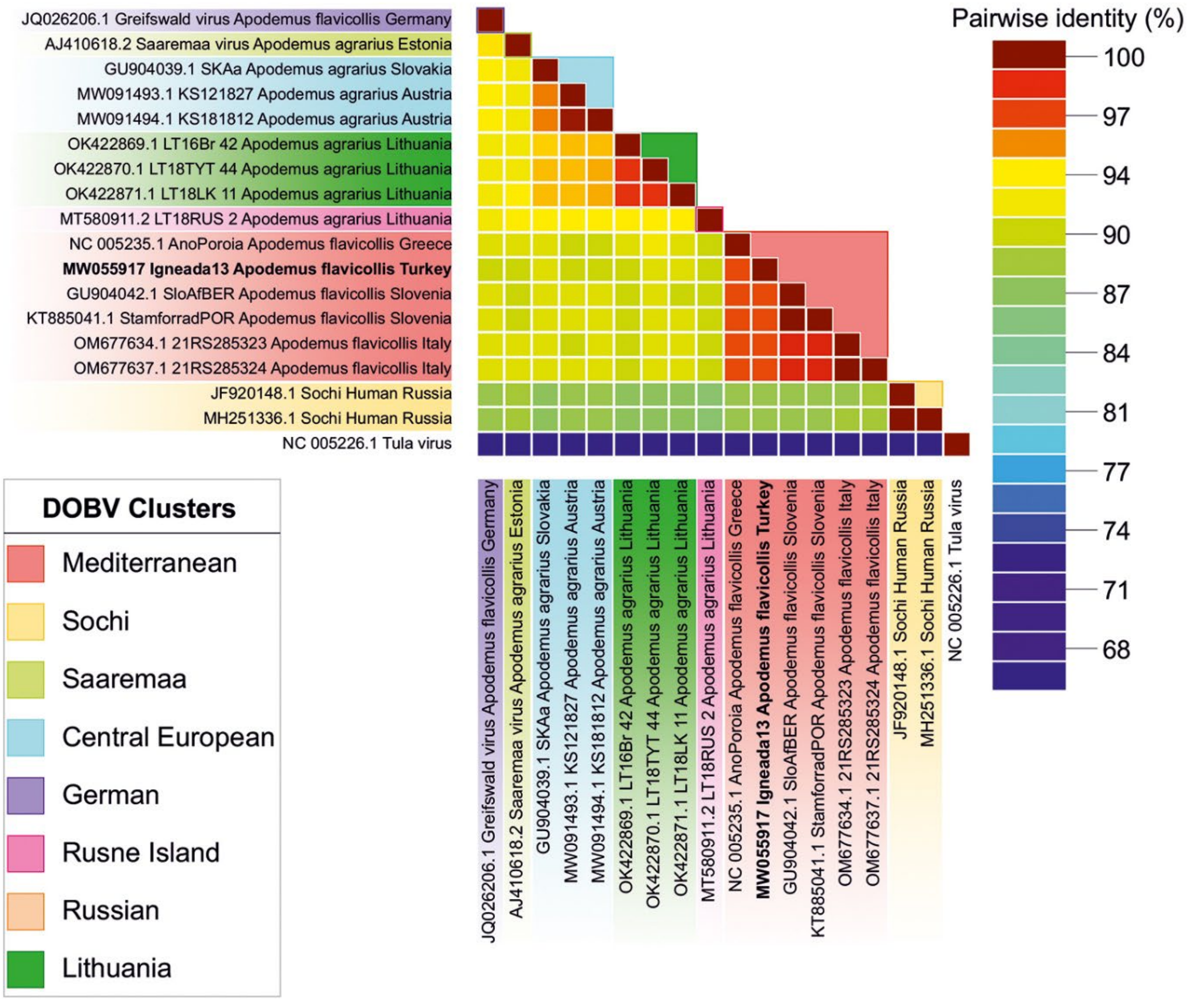

Appendix Figure 2. The pairwise identity matrices of A) S, B) M, and C) L segments showed correlating geographical clustering and different divergence among segments.

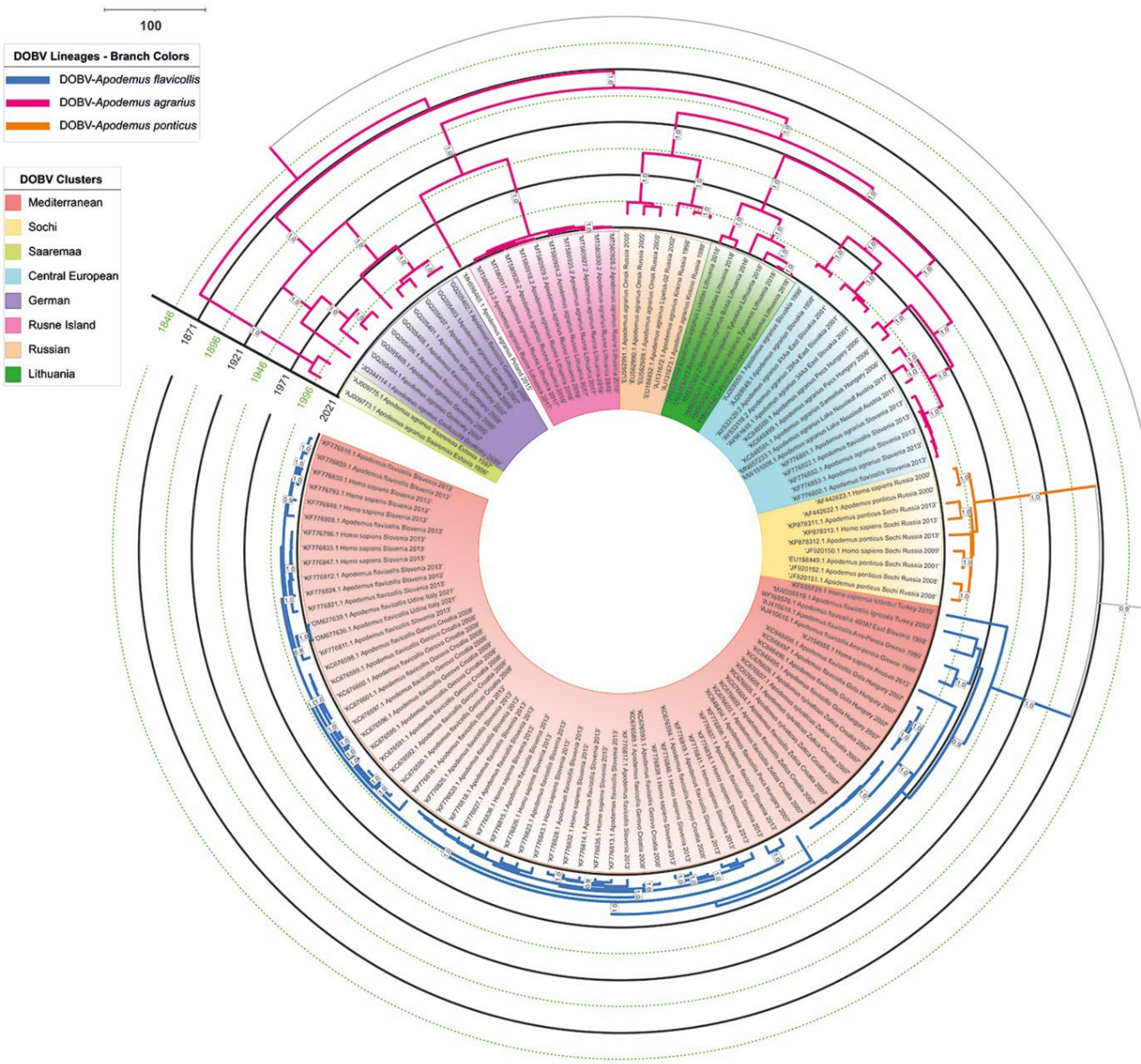

Appendix Figure 3. Bayesian maximum clade credibility (MCC) tree from phylogeographic reconstruction with total of 127 taxa. The MCC tree showed similar results as minimum spanning trees and ML trees by showing host-dependent lineage separation followed by geographic cluster separation. One sequence from Poland wasn't involved in any cluster under DOBV-*Apodemus agrarius* lineage due to insufficient data availability to make more detailed cluster hypothesis from that specific region.

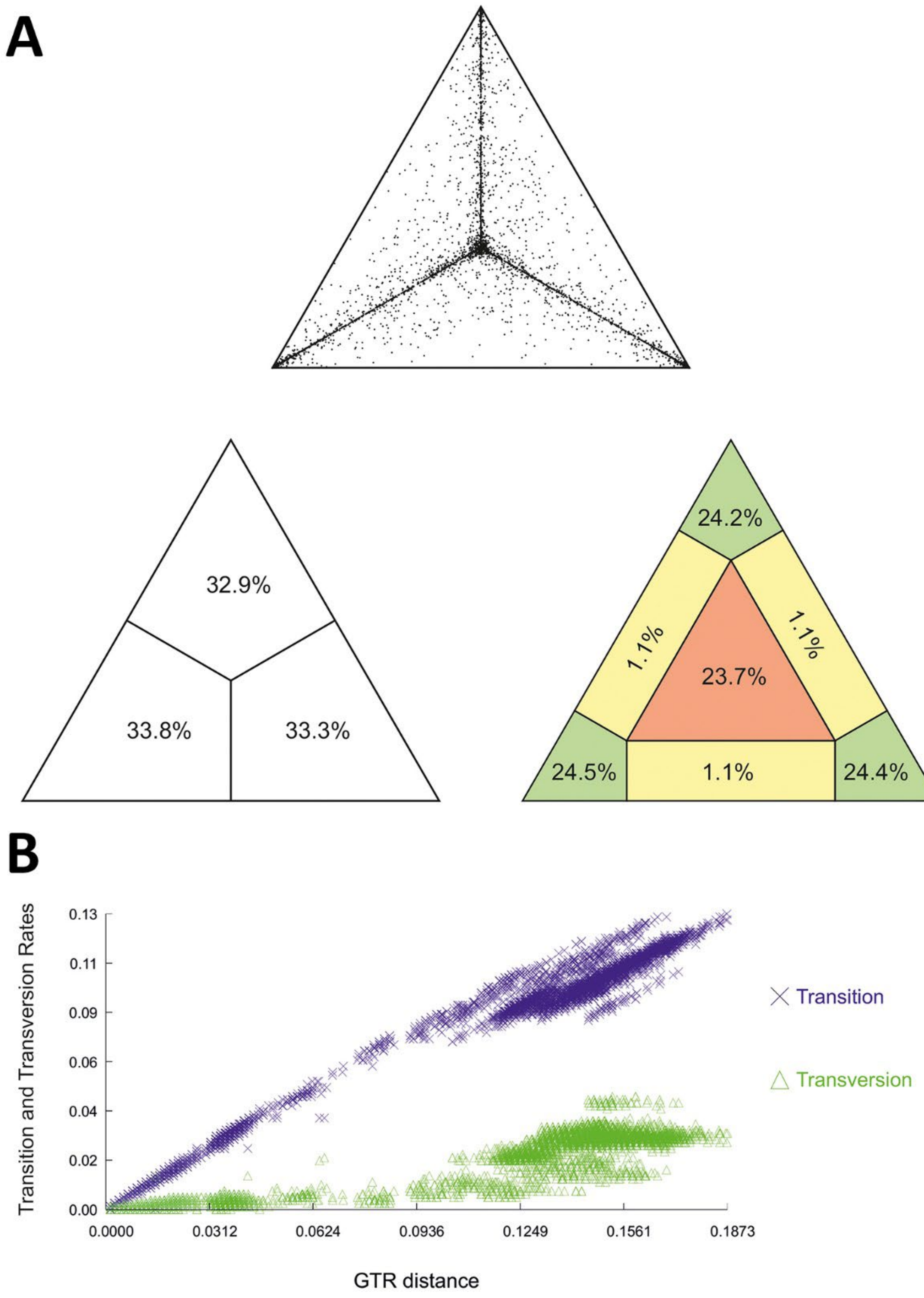

Appendix Figure 4. The dataset phylogenetic information testing. (A) 73.1% of the quartets in the likelihood mapping placed at the corners of the triangle by being fully resolved, yet 23.7% of the quartets, as a big proportion, placed at the middle triangle and formed phylogenetically uninformative part of the assessment. (B) The molecular saturation of the dataset was none to low which provided the sight of some analysis estimates being underestimated.
